# Brain-computer interface on wrist training with or without neurofeedback in subacute stroke: a study protocol for a double-blinded, randomized control pilot trial

**DOI:** 10.3389/fneur.2024.1376782

**Published:** 2024-07-31

**Authors:** Myeong Sun Kim, Hyunju Park, Ilho Kwon, Kwang-Ok An, Joon-Ho Shin

**Affiliations:** ^1^Translational Research Center for Rehabilitation Robots, National Rehabilitation Center, Ministry of Health and Welfare, Seoul, Republic of Korea; ^2^Department of Rehabilitative and Assistive Technology, National Rehabilitation Center, Rehabilitation Research Institute, Ministry of Health and Welfare, Seoul, Republic of Korea; ^3^Department of Healthcare and Public Health Research, National Rehabilitation Center, Rehabilitation Research Institute, Ministry of Health and Welfare, Seoul, Republic of Korea; ^4^Department of Rehabilitation Medicine, National Rehabilitation Center, Ministry of Health and Welfare, Seoul, Republic of Korea

**Keywords:** stroke rehabilitation, brain machine interface, brain-computer interface, randomized clinical trial, clinical trial protocol

## Abstract

**Background:**

After a stroke, damage to the part of the brain that controls movement results in the loss of motor function. Brain-computer interface (BCI)-based stroke rehabilitation involves patients imagining movement without physically moving while the system measures the perceptual-motor rhythm in the motor cortex. Visual feedback through virtual reality and functional electrical stimulation is provided simultaneously. The superiority of real BCI over sham BCI in the subacute phase of stroke remains unclear. Therefore, we aim to compare the effects of real and sham BCI on motor function and brain activity among patients with subacute stroke with weak wrist extensor strength.

**Methods:**

This is a double-blinded randomized controlled trial. Patients with stroke will be categorized into real BCI and sham BCI groups. The BCI task involves wrist extension for 60 min/day, 5 times/week for 4 weeks. Twenty sessions will be conducted. The evaluation will be conducted four times, as follows: before the intervention, 2 weeks after the start of the intervention, immediately after the intervention, and 4 weeks after the intervention. The assessments include a clinical evaluation, electroencephalography, and electromyography using motor-evoked potentials.

**Discussion:**

Patients will be categorized into two groups, as follows: those who will be receiving neurofeedback and those who will not receive this feedback during the BCI rehabilitation training. We will examine the importance of motor imaging feedback, and the effect of patients’ continuous participation in the training rather than their being passive.

**Clinical Trial Registration:** KCT0008589.

## 1 Introduction

Patients with stroke commonly face restrictions in their daily activities and social engagement because of impaired upper limb function, resulting in a decline in their overall quality of life (1). Rehabilitation enables the restoration of motor functions (2). Enhanced post-stroke function has been reported to result from recent approaches that incorporate more intensive and repetitive training, realistic and contextually relevant exercises, motivation, and active participation. These methods have been reported to be more effective than conventional and passive interventions (3, 4), and they use brain-computer interface (BCI) technology, demonstrating its efficacy in stroke rehabilitation regarding clinical functionality and neurophysiological changes (5, 6).

BCI acquires physiological signals, including brain waves, to accurately assess a patient’s intent and condition. Multimodal sensory stimulation has been adopted as a therapy involving mirror therapy, action observation, motor imagery (MI), and virtual reality (VR) training to acquire motor-relevant signals (7, 8). Meanwhile, feedback is needed to complete the motor performance loop and finally to accomplish plastic changes in the motor pathway, including creating alternative pathways or restoring the original pathway (9). Additionally, real-time feedback during the therapeutic process could augment this plastic change (10). Most importantly, the BCI was successfully operated by the closed loop connecting the motor intention to feedback, indicating the actual execution of motor tasks. This connection is necessary to effectively reorganize the specific neural circuits targeted in training, ultimately leading to functional improvement.

Earlier studies have shown the importance of the close relationship between motor intention and feedback (11–13). However, there is a lack of studies demonstrating the superiority of BCI operated using MI-contingent feedback (real BCI) over BCI operated using MI-irrelevant feedback (sham BCI). Two studies have only proven the superiority of real BCI over sham BCI. Ramos-Murguialday et al.’s study using BCI-driven finger orthosis (14) revealed a noteworthy enhancement in motor function, evident in the upper limb Fugl–Meyer assessment (FMA) scores of the real-BCI group compared to the sham-BCI group. In a separate study using FES as a feedback modality, Biasiucci et al. (15) also showed significant disparities in FMA improvement and wrist extensor muscle strength between real and sham BCI, favoring real BCI. However, these two studies enrolled only patients with chronic stroke. Thus, the favorable effects of real BCI were not confirmed in the subacute phase of stroke. Frolov et al. exhibited enhancements within a real-BCI group in patients with subacute and chronic stroke. However, they did not directly compare real-BCI and sham-BCI (16). Moreover, they did not enroll patients with subacute and chronic phase stroke separately at the beginning of the study. However, they analyzed them by categorizing them into subacute and chronic groups after the termination of the study.

A recent study demonstrated that functional recovery predominantly occurs within the first 2–3 months after stroke (17). Patients who undergo early rehabilitation therapy after a stroke tend to exhibit better outcomes, with significant recovery occurring within the early phase after stroke, such as the subacute phase (18, 19). Similarly, patients with subacute stroke benefit from training with a BCI. Wu et al. (20) demonstrated that the BCI significantly enhanced upper limb motor function and reorganized brain functional networks. Therefore, it needs to be verified whether real BCI is superior to sham BCI in the subacute phase of stroke. In this study, we aim to compare the effects of real and sham BCI on motor function and brain activity among patients with subacute stroke with weak wrist extensor strength.

The recently launched RecoveriX PRO system is a BCI-based rehabilitation training system for patients with stroke. The system measures the sensorimotor rhythm generated in the motor cortex during the imagined movement without external physical movement. Subsequently, it provides visual feedback through VR and induces movement through functional electrical stimulation (FES) (21). Importantly, feedback is provided only when the patient intends to move, encouraging active participation and enhancing neuroplasticity and the brain’s ability to self-restructure and reorganize in response to external stimuli. We will utilize RecoveriX PRO system for our proposed study to compare the effects of BCI on motor function and brain activity among patients with subacute stroke with weak wrist extensor strength.

## 2 Methods and analysis

### 2.1 Trial design

**Figure 1:**
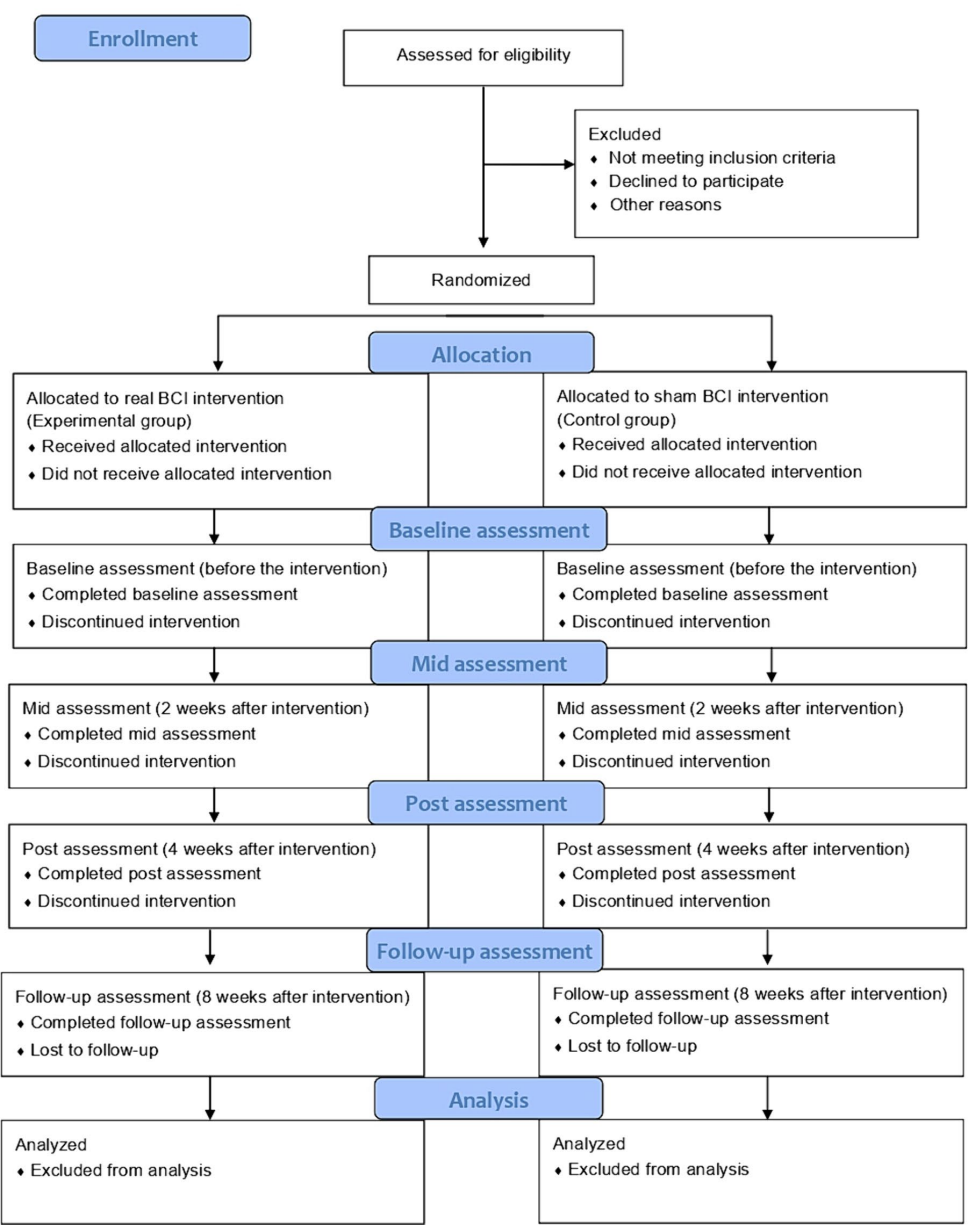
CONSORT flow diagram.

This intervention will be performed as a double-blind, parallel-group (1:1 ratio), randomized controlled trial. In addition, it will be conducted in an independent and quiet environment, where maximum concentration can be achieved with the BCI. The therapist will always be beside the participant in preparing for any issues during the intervention. We will assess the measures to determine the extent of improvement provided by the intervention over time. The CONSORT flow diagram is shown in Figure 1.

### 2.2 Participants

This study will be conducted at the National Rehabilitation Center in the Republic of Korea, and the experiment will be performed at the same center. The Institutional Review Board of the Rehabilitation Hospital approved this study (IRB no. NRC-2023-02-013). This study will involve human participants and aims to investigate the effects of BCI on subacute stroke. The study will be conducted in accordance with ethical statements and guidelines for human research. All participants who agree to participate after understanding the contents of this research will provide written informed consent before enrollment. This study was registered with CRIS (cris no. KCT0008589).

#### 2.2.1 Inclusion criteria

The inclusion criteria are as follows: (1) hemiplegic participants secondary to a first-ever stroke with lesions confined to one hemisphere; (2) subacute stroke, which is defined as motor dysfunction occurring within 1 week to 6 months after stroke onset; (3) Medical Research Council (MRC) scale of affected wrist extensor muscle strength ≦ 2; (4) age > 19 years; and (5) a patient who has the cognitive ability to understand and follow the instructions of the researcher.

#### 2.2.2 Exclusion criteria

The exclusion criteria are as follows: (1) factors impeding recording of electroencephalogram (EEG) signals such as skin infections, scalp wounds, and the presence of metal implants under electrodes; (2) spasticity of wrist flexor, scoring ≥2 on the modified Ashworth scale; (3) cognitive impairments or severe aphasia hindering the ability of the participant to understand and follow the instructions during the research; (4) presence of a neurological or psychiatric disorder other than stroke; (5) musculoskeletal problem or severe pain of the affected upper limb impeding intervention; (6) hemispatial neglect; (7) patients with epilepsy.

### 2.3 Interventions

#### 2.3.1 BCI-FES system

**Figure 2:**
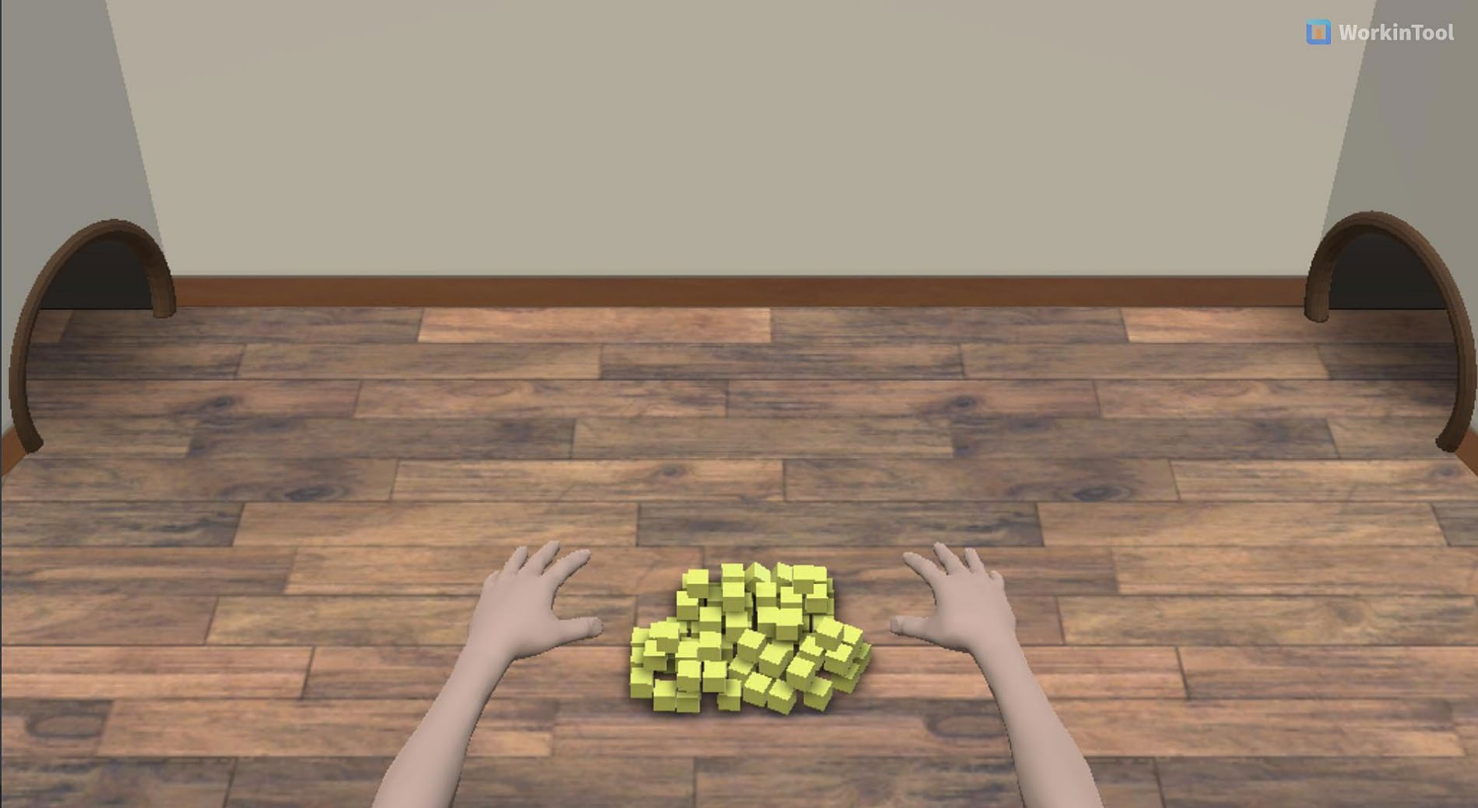
Virtual avatar of RecoveriX PRO intervention.

**Figure 3:**
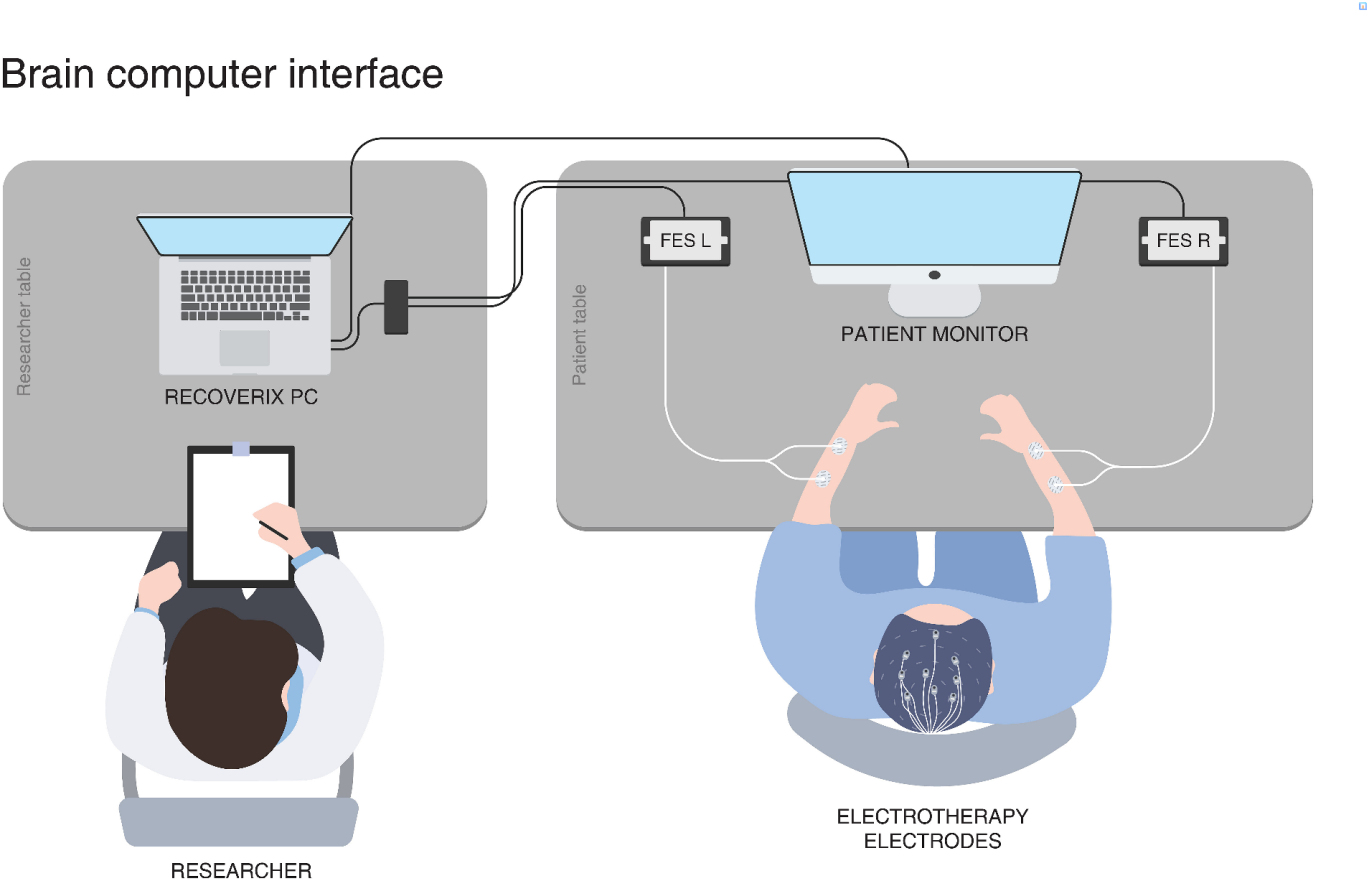
RecoveriX PRO study setting.

The BCI intervention tool employed in this study is the RecoveriX PRO, a noninvasive neurofeedback-based stroke rehabilitation system developed by g.tec Medical Engineering GmbH in Austria. The system integrates an EEG, FES, and a computer screen projecting virtual hands. The EEG signals are recorded using a g. nautilus PRO FLEXIBLE (g.tec Medical Engineering GmbH, Austria). There are 16-channel systems (FC3, FCz, FC4, C5, C3, C1, Cz, C2, C4, C6, CP3, CP1, CPz, CP2, CP4, and Pz) that capture data from specific locations according to the internationally recognized 10–20 systems. The g. Nautilus PRO FLEXIBLE allows plug-in electrode strands with Strand 16 g SCARABEO (g.tec Medical Engineering GmbH, Austria) gel-based EEG electrodes. The ground and reference electrodes are positioned on the forehead (FPz) and right earlobe. Spatial filtering, which employs the common spatial pattern method, aims to maximize the variance for one category of MI and minimize the variance for the other, followed by linear discriminant analysis for classification. FES electrodes are placed over the wrist extensors of both forearms, with a frequency of 50 Hz and a rectangular pulse width of 300 μs using g.Estim FES devices (g.tec medical engineering GmbH, Austria). The current amplitude will be customized to ensure contraction of the wrist extensor muscles on the affected side without pain, spasms, or fatigue. In the virtual avatar format, both hands and arms are placed on a table with a cheese-shaped cube positioned between them on the screen (Figure 2). Visual feedback is provided through a game where the objective is to compete with a mouse to protect a food item (cheese). At the start of each session, 80 pieces of cheese are placed between the two virtual arms. During each exercise, the mouse emerges from either the right or left corner of the room (corresponding to the wrist that must be moved) and is situated near the pile of cheese pieces. The RecoveriX PRO system integrates, analyzes, and recognizes EEG signals associated with MI, activating the FES system when the participants imagine movements on the instructed side. The RecoveriX PRO implements paired associative stimulation, connecting recorded MI with output feedback through visual feedback (virtual avatar wrist extension) and FES for wrist extension. Consequently, the system establishes a closed loop between brain signals during the desired MI and contingent visual and proprioceptive feedback, aiding patients in learning to imagine or perform the desired movements. The study setting is illustrated in Figure 3.

#### 2.3.2 Intervention description

**Figure 4:**
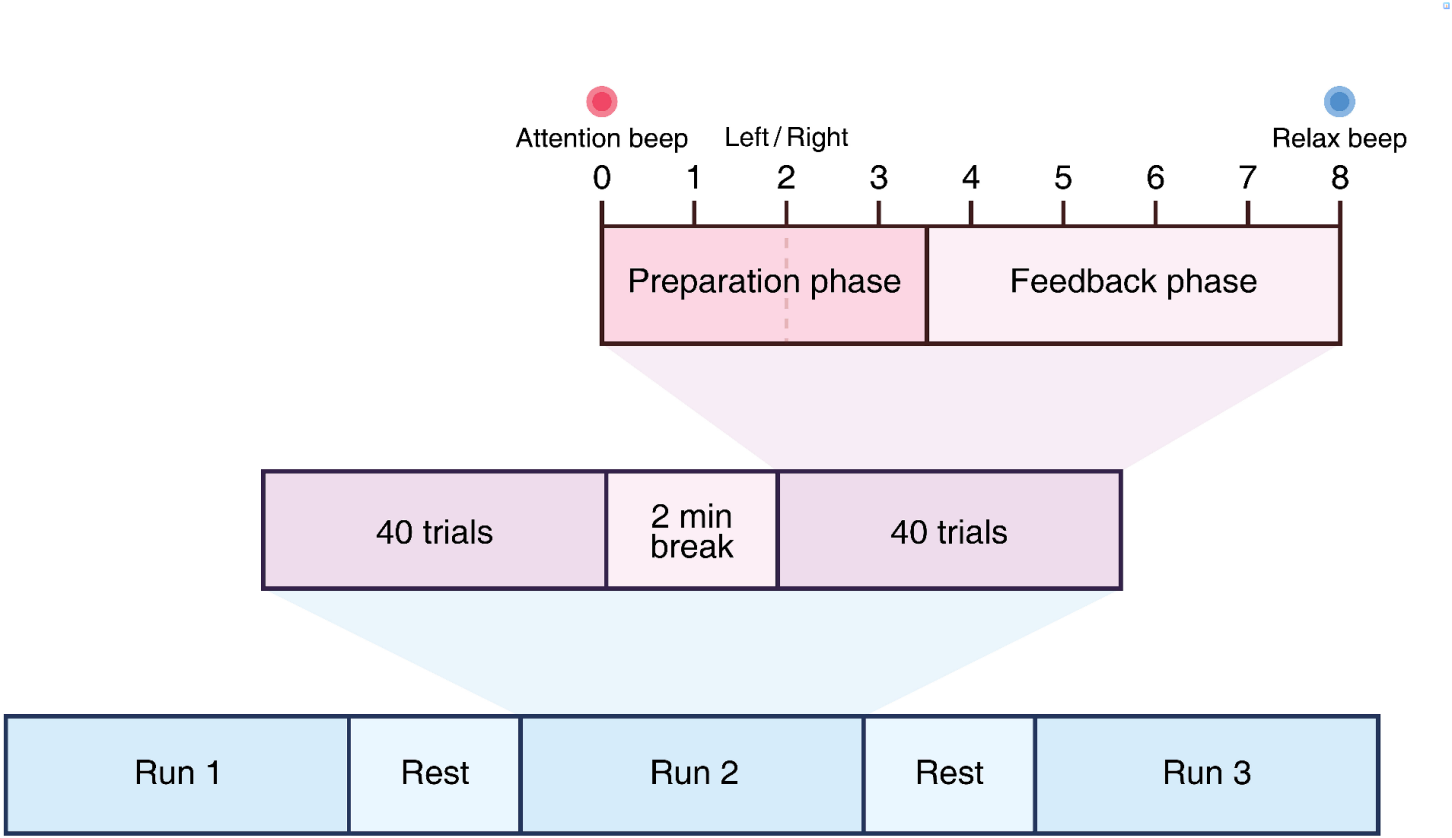
RecoveriX PRO rehabilitation procedure.

The RecoveriX PRO interventions involve 240 trials of MI tasks for both hands, which are further classified into three runs of 80 trials each. Each run consists of two sets of 40 trials, with a 2-min break between consecutive sets and a 3-5-min break between runs. A trial is initiated with an attention beep at 0 s. The corresponding verbal instructions (“left” or “right”) in the participants’ native language will be presented at 2 s. The MI task involves imagining wrist dorsiflexion based on the system’s instruction, which indicates “left” or “right” in a random order. During the feedback phase (from 3.5 s to 8 s), the FES and virtual avatar are activated. The interventions comprise two types of runs: calibration and training. The primary distinction is the feedback phase (MI-contingent feedback vs. MI-independent feedback). In the calibration run, feedback is consistently activated regardless of the MI. Contrarily, in the training run, feedback is only activated when the BCI system detects the MI of the correct hand. The feedback is updated five times per second. More importantly, the calibration run is combined with FES in each session. The relaxation signal is presented at 8 s, and the task period (MI) lasts 6 s. RecoveriX PRO rehabilitation procedure is presented in Figure 4.

All participants will undergo 20 sessions of 60-min BCI intervention (real BCI intervention or sham BCI), 5 days a week for 4 weeks, administered by research physical therapists. The real BCI intervention session includes one calibration run and two subsequent training runs in which participants will receive an MI-contingent feedback-based BCI intervention. In contrast, the sham BCI intervention session comprises three consecutive calibration runs without any training runs. Thus, participants will receive MI-independent feedback (FES and visual feedback) on either hand, regardless of their MI. The participants will perform the MI task seated while wearing an EEG cap and observing the virtual avatar’s forearm and hand on a screen. The intervention and assessments will be conducted in an independent and quiet research room within a rehabilitation hospital, enabling a focused approach toward the task during the intervention.

Additionally, all participants will receive 30 min of conventional therapy for the affected upper limb 5 days a week for 4 weeks. The progress or response to therapy will be assessed after each session, and the results will be subsequently calculated. The accuracy plot is a metric for distinguishing left and right movements, indicating how well the participant performed MI during exercise. If the accuracy plot fails to surpass a significant threshold, an additional calibration exercise will be conducted to facilitate the smooth progression of the training mode.

### 2.4 Outcomes

The evaluation will be conducted four times, as follows: before the intervention (T0), 2 weeks after the start of the intervention (T2), immediately after the end of the intervention (T4), and 4 weeks after the end of the intervention (T8). There are three outcome measures, as follows: primary, self-reported, and neurophysiological. The SPIRIT participant timeline is shown in Table 1.

**Table 1:** SPIRIT overview measurements and time points.

	Study period								
		Enrolment	Allocation	Baseline	Intervention	2 weeks	Intervention	4 weeks	8 weeks
Time points				T 0	Week 0–2	T2	Week 2–4	T4	T8
**Enrolment**									
Eligibility screening		Χ							
Informed consent		Χ							
Allocation			Χ						
**Intervention**									
Experimental					Χ		Χ		
Control					Χ		Χ		
**Assessments**									
Primary	MRC			Χ		Χ		Χ	Χ
	FMA			Χ		Χ		Χ	Χ
	AROM			Χ		Χ		Χ	Χ
	MAS			Χ		Χ		Χ	Χ
Self-report	SIS			Χ		Χ		Χ	Χ
	SRMS			Χ		Χ		Χ	Χ
	IMI							Χ	
	SoO, SoA							Χ	
Neurophysiology	EEG			Χ		Χ		Χ	Χ
	MEP			Χ		Χ		Χ	Χ

MRC, Medical Research Council; FMA, Fugl-Meyer Assessment; AROM, Active Range of Motion; MAS, Modified Ashworth scale; SIS, Stroke Impact Scale; SRMS, Stroke Rehabilitation Motivation Scale; IMI, Intrinsic Motivation Inventory; SoO, Sense of Ownership; SoA, Sense of Agency; EEG, Electroencephalography; MEP, Motor-Evoked Potential.

#### 2.4.1 Primary outcomes

The MRC scale will be used to test the wrist strength (22). The MRC score ranges from 0 to 5 as follows: grade 0, no visible contraction; grade 1, visible contraction without movement of the limb; grade 2, movement of the limb but not against gravity; grade 3, the movement against gravity over (almost) the full range; grade 4, the movement against gravity and resistance; and grade 5, normal.

#### 2.4.2 Secondary outcomes

We will use the upper extremity of the FMA to assess the motor function of the upper extremity. The FMA evaluates body function encompassing a wide range of assessments. It is highly recommended for evaluating upper extremity function and activity in both research and clinical practice (23). Scores on this assessment range from 0 to 66. We will use the Modified Ashworth scale (MAS) to evaluate muscle spasticity (24). It consists of six points, as follows: 0, no tonus increase; 1, the presence of a catch-and-release feeling at the end of the range of motion or a slight tonus increase in character with minimal resistance; 1 +, there is a slight increase in muscle tone observed through minimal resistance throughout less than half of the joint range of motion; 2, muscle tone is increased throughout the range of motion of the whole joint, but the joints can be moved easily; 3, there is significant tonus increase, which makes passive movement difficult; and 4, the affected parts are rigid in flexion and extension. In addition, we will evaluate the active range of motion in the wrist extension/flexion. The Stroke Impact Scale (SIS) serves as a self-report evaluation for measuring stroke outcomes and is utilized for assessing Health-Related Quality of Life. It assesses eight domains (strength, hand function, mobility, activities of daily living, emotion, memory, communication, and social participation) on a 5-point scale (25). The Korean Version of the Stroke Rehabilitation Motivation Scale (K-SRMS) measures the degree of motivation in patients with stroke during rehabilitation interventions (26). It includes 24 items, and the responses to each item are measured on a scale of 1–5. It is categorized into seven subscales: extrinsic motivation (EM)-introjected (motivation from external factors leading to internal pressures, such as guilt), EM-regulation (motivation from external pressures or rewards, such as praise from others), EM-identification (motivation from activity participation for personal growth), amotivation (AM; absence of motivation), intrinsic motivation (IM)-knowledge (motivation from knowledge gained from the activity), IM-stimulation (motivation from enjoyment or pleasure of an experience), and IM-accomplishment (motivation from personal satisfaction with the activity accomplishment). We will assess the sense of ownership (SoO) and agency (SoA) using questionnaires to understand the experience of BCI in patients. SoO describes the feeling of mindedness toward one’s body parts, feelings, or thoughts. In contrast, SoA refers to the experience of initiating and controlling an action. SoO and SoA are utilized to assess users’ sense of ownership and agency over a virtual limb, as well as to regulate the movements of an artificial hand (27). SoO will be assessed using an 8-statement questionnaire and SoA, using a 16-statement questionnaire; of the 16 questions, eight belong to the FES agency, and the remaining eight belong to the VR agency. It is reported on a 7-point Likert scale ranging from −3 (“totally disagree”) to 3 (“totally agree”), whereby 0 indicates neither agreement nor disagreement (“neutral”). The IMI is a multidimensional measurement device intended to assess participants’ subjective experiences related to a target activity in laboratory experiments (28). It assesses seven subscales: (1) participants’ interest/enjoyment; (2) perceived competence; (3) effort; (4) value/usefulness; (5) felt pressure and tension; (6) social relatedness; and (7) perceived choice while performing a given activity. It is composed of a 45-item questionnaire 7-point Likert scale (ranging from “completely disagree” = 1 to “completely agree” = 7). The SoO and SoA questionnaires and IMI will be assessed only at immediately after the end of the intervention (T4).

#### 2.4.3 Neurophysiological outcomes

We will acquire brain signals before and after the intervention to compare the brain activity. Electroencephalography (EEG) signals will be recorded using a 32-channel g.Nautilus system (g.tec Medical Engineering). The electrodes will be placed according to the extended 10–20 international system. The ground channel will be located on the forehead, and the reference channel will be in the right earlobe. The participants will be seated in a comfortable chair. The session will start with preparing and placing electrodes (lasting approximately 30 min) and will continue with resting-state recordings (20 min). We will instruct all participants not to think about anything. We will acquire brain signals with the participants’ eyes closed and eyes open at rest for 5 min twice (20 min).

Cortical excitability will be measured using transcranial magnetic stimulation (TMS) (MagPro stimulator, MagVenture, Lucernemarken, Denmark) at four time points (T0, T2, T4, and T8). During the measurements, the participants will be seated in a comfortable recliner and will hold their hands on their laps in a supine position. The participants will remain silent during the study to avoid speech-induced modulation of cortical excitability. The participants will also be monitored for drowsiness and will be asked to keep their eyes open throughout the motor-evoked potentials (MEP) measurements. A figure-of-8 coil will be employed to stimulate the motor cortex, orienting the coil handle 45° posterior to the midline to ensure perpendicular electromagnetic current flow to the central sulcus (29). The coil will be moved over the scalp in 1-cm increments. For the electromyographic (EMG) signal recording, an active surface electrode will be attached to the contralateral first dorsal interosseous muscle, while reference and ground electrodes will be placed on the index finger’s proximal interphalangeal joint and over the wrist, respectively. Relaxation of the measured muscles will be controlled by continuous visual EMG monitoring. EMG signals will be captured in terms of amplitude and frequency. The “hot spot,” identified as the scalp location yielding the largest MEP amplitude with the lowest stimulation intensity, will be determined (30).

We will assess the resting motor threshold (RMT), defined as the lowest stimulator output intensity eliciting MEPs with an amplitude of at least 50 microvolts in at least five out of 10 consecutive trials (30). We will also examine the MEPs, an electrical signal recorded from the descending motor pathways or muscles following the stimulation of motor pathways within the brain RMT (%). MEP amplitude will be evaluated at 120% of the TMS intensity required to elicit the RMT, and the average peak-to-peak amplitudes of MEPs from 10 consecutive sweeps will be recorded.

We will employ paired-pulse stimulation to assess intracortical inhibition (ICI) and facilitation (ICF) (31). We will initially apply a conditioning stimulus (subthreshold; set at 80% of RMT) and a test stimulus (suprathreshold, set at 120% of RMT) on the affected motor cortex. The MEP values obtained with an inter-stimulus interval of 3 ms will be used to measure ICI and those obtained with an inter-stimulus interval of 10 ms will serve as a measure of ICF. The raw average values for both ICI and ICF will be recorded. Furthermore, we will calculate the ICI and ICF percentages, normalized based on the average MEP obtained with the unconditioned stimuli (set at 120% of the RMT). ICF is a process in which the activity of one neuron facilitates that of another, whereas ICI involves a presynaptic neuron that inhibits the firing of another neuron.

### 2.5 Sample size

We were unable to perform a sample size calculation owing to the absence of a prior study that compared real-BCI and sham BCI using the identical BCI system. Consequently, we have established a sample size of 20 for each arm, adhering to the minimum number suggested for pilot trials (32).

### 2.6 Recruitment

People will be recruited at the National Rehabilitation Center according to the inclusion and exclusion criteria.

### 2.7 Assignment of interventions: allocation and blinding

Forty patients with stroke will be enrolled in this study. The random number table, which assigned the participants to either the real-BCI group or the sham-BCI group, was generated by PASS in the order of recruitment of participants. An external researcher will create a random number table and maintain it until the end of the study. The assignment will proceed by opening an opaque envelope containing a random number table in the order of participant recruitment. All participants will be blinded without knowledge of the group they will be assigned to.

### 2.8 Data collection and management

#### 2.8.1 Plans for assessment and collection of outcomes

All the participants will complete a baseline assessment before commencing the intervention. Then, assessments will be performed 2 weeks after starting the intervention (after the 10th session of intervention) and after the completion of the intervention (after the 20th intervention session). A follow-up test will be performed 4 weeks later. An experienced research physical therapist will conduct all clinical assessments and EEG testing, and physiatrists will perform TMS measurements. The assessments will be conducted without the participants knowing their assigned group. All data in this study will be recorded and managed using the data management software in Microsoft Excel. All researchers will use an encrypted computer to record and save data to ensure data security, with backup data to prevent data loss.

### 2.9 Plans to promote participant retention and complete follow-up

Patients at our hospital can be hospitalized for up to 3 months, providing sufficient time for follow-up evaluation (T8). Follow-up evaluation will be conducted after finishing the intervention.

### 2.10 Data management

All data will be stored electronically. The approved case report form (CRF) will be saved by coding at the end of the patient evaluation. The evaluator will conduct a clinical evaluation on paper and transfer it to the CRF. In addition, in the case of evaluation using medical equipment, such as EEG and MEP, files will be created and stored for each patient ID. Monitors, inspection personnel, and the National Rehabilitation Center Clinical Trial Review Board will not directly view or submit research-related data to verify the reliability of the procedure and data of this study within the scope stipulated by the relevant regulations, without infringing on the confidentiality of the research participants. In addition, the analysis will be conducted using only the study ID and raw research data. Personal identifiable information will be securely destroyed after being stored for 3 years after completion of the study.

### 2.11 Statistical methods

A repeated-measures analysis of variance at the time level (T0, T2, T4, and T8) will be applied to examine whether primary, secondary, and neurophysiology outcomes improved after the intervention. Paired t-tests will be used as post-hoc tests to examine significant changes in different combinations of time points for the scores. The normality of the data will be checked using Kolmogorov–Smirnov tests, and the results will show whether the data are normally distributed. The Bonferroni correction will be used for multiple comparisons.

The Friedman test will be performed for within-group comparisons between time points for non-normally distributed data. The Wilcoxon signed-rank test will also be used for within-group comparisons at each point in time. The resting-state EEG data (with eyes closed) will be first preprocessed to analyze the changes in functional connectivity in the motor area before and after the intervention. The raw EEG data will first be filtered using a 3rd-order Butterworth filter with 1 and 50 Hz cutoff frequencies. Subsequently, it will be divided into 1-s epochs without overlaps. Epochs showing large artifacts, detected using a signal threshold of ±120 μV, will be removed. Next, 30 artifact-free epochs will be randomly chosen for each participant. Partial directed coherence (PDC), a representative effective functional connectivity measure, will be used to evaluate changes in directed functional connectivity from the premotor area to the motor area before and after the intervention (15, 33). Based on the previous study (15), two frequency bands, μ (10–12 Hz) and β (18–24 Hz) bands, will be used for the calculation of PDC. The PDC values will be normalized to a range of 0–1 and then averaged across each participant. Ten consecutive MEPs will be recorded for each stimulus intensity to measure the changes in corticomotor function. These measurements will determine each subject’s average ICI and ICF responses by calculating the mean of 10 trials of paired-pulse TMS. Statistical analyses will be performed using SPSS 21.0 (IBM SPSS Statistics, NY, United States), with the significance level set at *p* < 0.05.

## 3 Discussion

Several systematic reviews on BCI have reported clinical and functional improvements and neurophysiological changes (5, 6, 34). These findings indicate that the proposed intervention method promotes optimal functional reorganization during the recovery process of the affected limb. However, it is essential to verify each BCI system, especially the working of MI-contingent feedback, because the communication of MI and neurofeedback is a core feature of BCI. The present study aims to verify the MI-contingent feedback of a commercially available BCI system, RecoveriX PRO. Considering the lack of commercially available BCI rehabilitation systems for patients with stroke, this verification could be helpful for real clinical settings. Additionally, there is a shortage of studies, which have verified this MI-contingent BCI system in patients in the subacute phase of stroke. Therefore, this study may accelerate the use of a BCI system in general stroke rehabilitation settings, not only limited to a research setting.

The strengths of our study include the intention to recruit a larger sample size than that of previous studies, particularly focusing on patients in the subacute stage of stroke. In addition, we aim to enhance the interpretation of the outcome measures by employing a diverse set of evaluation tools. Our study seeks to incorporate a variety of assessments, including EEG and MEP, that have not been comprehensively utilized in prior investigations. This approach will allow for a more comprehensive evaluation of neuroplasticity from the cerebral cortex to the end-effector. Furthermore, our study aims to measure the extent of BCI experience, motivation, and quality of life through subjective questionnaires. Overall, we believe that these data will serve as a foundation for demonstrating the comprehensive impact of BCI.

However, an expected limitation of this study is the potential occurrence of fatigue and tiredness because of the daily sessions conducted five times a week for 1 h each. This type of fatigue is commonly observed in BCI studies. If participants report feeling fatigued or finding the task challenging, we will proceed only after ensuring that they take sufficient rest. In addition, therapists will consistently motivate the patient throughout the process. Further, after each run, they will display the results provided by the RecoveriX PRO to encourage and maximize the patients’ motivation. Unlike traditional BCIs, our study employs a virtual reality-based game task. We believe that the game will enhance motor performance, as BCI participation is facilitated by motivation and active engagement (35). Moreover, feedback provided by the game plays a crucial role in sustaining patient concentration (36). Another issue is the sample size, which may not be sufficient to fully capture the variability in the population, potentially limiting the generalizability of the findings. However, compared to previous studies, our sample size cannot be considered small. We will recruit 20 patients per arm. Therefore, should be able to minimize the likelihood of these limitations.

The protocol used in this study can serve as a foundation for future basic research in the clinical setting of BCI interventions. Furthermore, it aims to stimulate the advancement of foundational research on MI-contingent feedback for BCI interventions in the subacute stroke domains. If these aspects are successful, BCI may play an important role in stroke rehabilitation in the future.

## 4 Conclusion

We hypothesize that contingency between MI-related neural correlates and rich sensory feedback is essential in closed-loop BCI systems for functional improvement and neural plasticity. Therefore, we aim to investigate whether differences in function and neural plasticity arise from the close contingent connection between MI-induced brain activity and the consequent feedback. We will compare the effects of the BCI system operated by MI-contingent feedback (real-BCI) versus MI-independent feedback (sham-BCI) on distal upper limb function and brain activity in patients with subacute stroke.

## 5 Ethics and dissemination

On the consent form, participants are asked whether they agree to the use of their information for research. The researchers will ensure that they will protect the confidentiality of all personal information obtained during this study and that the form they sign will ensure this. When disclosing personal information obtained from this research to a journal or conference, their names and other personal details will not be included. However, personal information may be provided if required by law. In addition, monitors, inspection personnel, and the National Rehabilitation Center Clinical Trial Review Board may directly view or submit research-related data to verify the reliability of the research procedure and data within the scope of the relevant regulations without infringing on the confidentiality of the research participants. By signing this agreement, the patient will be deemed to have knowledge of its contents and agree to comply with it. The results of this research will be published in academic journals and journal papers, and the institution’s reports will also be published.

## Author contributions

MK: Writing – review & editing, Writing – original draft, Visualization, Validation, Methodology, Formal analysis, Data curation, Conceptualization. HP: Writing – review & editing, Visualization, Validation, Methodology, Formal analysis, Data curation, Conceptualization. IK: Writing – review & editing, Visualization, Validation, Methodology, Formal analysis, Data curation, Conceptualization. K-OA: Writing – review & editing, Visualization, Validation, Methodology, Formal analysis, Data curation, Conceptualization. J-HS: Writing – review & editing, Validation, Supervision, Project administration, Methodology, Funding acquisition, Formal analysis, Data curation, Conceptualization.
